# An observational study of 110 elderly lithium-treated patients followed up for 6 years with particular reference to renal function

**DOI:** 10.1186/s40345-017-0089-1

**Published:** 2017-06-04

**Authors:** Alberto Bocchetta, Francesca Cabras, Martina Pinna, Antonio Poddighe, Claudia Sardu, Raffaella Ardau, Caterina Chillotti, Maria Del Zompo

**Affiliations:** 1 Unit of Clinical Pharmacology, Azienda Ospedaliero-Universitaria di Cagliari, “San Giovanni di Dio” Hospital, Via Ospedale 54, 09124 Cagliari, Italy; 2 Section of Neurosciences and Clinical Pharmacology, Department of Biomedical Sciences, University of Cagliari, Cagliari, Italy; 3 Neurology Outpatient Unit, Local Health Agency, Cagliari, Italy; 4 Department of Medical Science and Public Health, University of Cagliari, Cagliari, Italy

**Keywords:** Mood disorders, Bipolar disorders, Depression, Lithium, Elderly, Creatinine, Kidney, Lithium, Nephrotoxicity, Thyroid

## Abstract

**Background:**

Recent observational studies have focused on lithium treatment in the elderly, with particular reference to safety in terms of thyroid and renal functions. The purpose of this study was to compare the clinical characteristics of patients starting lithium treatment before (*N* = 79) or after (*N* = 31) the age of 65 years. Patients were followed up for 6 years with focus on renal function and prescription of levothyroxine and methimazole.

**Results:**

At baseline, median lithium serum concentration was 0.55 mmol/l. The estimated glomerular filtration rate was lower than 60 ml/min/1.73 m^2^ in 43 (39%) patients. In a multiple regression analysis controlling for age and gender, we found a significant effect of duration of lithium treatment on estimated glomerular filtration rate (−0.85 ml/min/1.73 m^2^ per year of prior exposure). The annual decline during follow-up was 2.3 ml/min/1.73 m^2^. Two patients were prescribed levothyroxine, and two were prescribed methimazole for the first time during follow-up.

**Conclusions:**

Median lithium serum concentration in this cohort of elderly patients with mainly bipolar disorders was lower than the therapeutic range indicated for younger adults. The decline in glomerular filtration rate may be accelerated by long-term lithium use. Thyroid and renal functions continue to require close monitoring throughout the course of lithium treatment.

*Trial registration* NP/2013/3836. Registered 24 June 2013

## Background

Reviews published over the last two decades continue to recommend lithium treatment in elderly patients with bipolar disorder (Young et al. 2004; Aziz et al. 2006; Shulman 2010). There is also evidence that lithium combined with antidepressants may be effective in the prophylactic therapy of elderly patients with unipolar depression (Wilkinson et al. 2002). Prescription patterns have shifted in favor of valproic acid over lithium for elderly patients with bipolar disorder, but this shift may not be completely evidence based (Shulman et al. 2003). Current national and international guidelines for bipolar disorder are incorporating emerging information, but the majority of guidelines do not have sections that pertain specifically to older adults (Dols et al. 2016). Research into the use of lithium in old age is very limited, because elderly subjects are frequently excluded from randomized clinical trials. One exception is the trial that has been performed specifically in late-life mania, but the key results for which are yet to be published (Young et al. 2010). Observational studies of elderly lithium patients continue to provide useful information, and recently, research has reported on thyroid and renal functions (Kraszewska et al. 2015; Permoda-Osip et al. 2016; Rej et al. 2013a, 2014a), and cognitive function and brain abnormalities (Gildengers et al. 2015; Zung et al. 2016) in these elderly populations.

Kraszewska et al. (2015) studied thyroid function in a cross-sectional sample of 66 patients with bipolar disorder receiving lithium over a period of between 10 and 44 years (mean age = 62 years). Of the 45 women in the sample, seven were receiving levothyroxine replacement therapy and an additional three had elevated thyrotropin concentrations. Therefore, some features of hypothyroidism occurred in 22% of their female sample.

Permoda-Osip et al. (2016) studied kidney, thyroid, and other organ functions after 40 years or more of lithium therapy in five patients (age range = 64–79 years). The three women in the sample had asymptomatic stage 2 chronic kidney disease (CKD) with an estimated Glomerular Filtration Rate (eGFR) ranging from 60 to 75 ml/min/1.73 m^2^. One male had stage 3 CKD (eGFR = 45–49 ml/min/1.73 m^2^), and the other had stage 2/3 CKD (59 ml/min/1.73 m^2^). One woman had severe thyroid dysfunction (Hashimoto’s disease) with extremely high levels of antithyroid peroxidase antibodies and antithyroglobulin antibodies and was receiving thyroxine. However, cognitive functions in all five patients were comparable to those in healthy people of similar gender, age, and education.

Rej et al. (2013b) conducted a retrospective study of 27 lithium-using geriatric patients (mean age = 81.6 years) with eGFR  ≤60 ml/min/1.73 m^2^ on ≥2 occasions within ≥3 months. Patients were divided into those who continued lithium use ≥2 years following CKD and those who discontinued lithium. Changes in eGFR between continuers and discontinuers after 60 months were not statistically significant. However, clinically important decreases in eGFR occurred in the majority of continuers but in none of the discontinuers.

A Canadian population-based cross-sectional study of 2480 lithium users aged 70 years or above measured prevalence of CKD, acute kidney injury, and nephrogenic diabetes insipidus. The 6-year prevalence rate of CKD was 13.9%. Lithium use for more than 2 years was one of the variables independently associated with CKD (Rej et al. 2014a).The purpose of the present study was to follow up a cohort of 110 cross-sectional lithium patients studied since 2010, when they were at least 65 years old. Our aims were (1) to compare patients who had become old during long-term lithium treatment with those who had been first prescribed lithium in old age, with particular regard to psychiatric diagnosis, and concurrent psychiatric and non-psychiatric medication; and (2) to follow up patients with regard to status of lithium treatment, and thyroid and renal functions.

## Methods

### Sample

Out of the 350 patients treated with lithium who had attended the Unit of Clinical Pharmacology, Azienda Ospedaliero-Universitaria, Cagliari, at least once between January and August 2010, we selected those born in 1945 or earlier (*N* = 110; 80 women, 30 men).

The Unit of Clinical Pharmacology has been one of the reference centers for lithium monitoring in the Cagliari area since its introduction in the 1970s. Lithium may be prescribed by any psychiatrists in this area, but elderly patients eligible for mood stabilizing treatment are often sent in by neurologists or general practitioners. Patients may continue to be followed by the referring specialist or may be managed by the lithium clinic staff alone.

The best estimate lifetime psychiatric diagnoses in the clinic are based on: direct interviews using the Schedule for Affective Disorders and Schizophrenia-Lifetime Version (SADS-L) (Endicott and Spitzer 1978), medical records, and information from relatives and referring specialists.

Once the initial treatment has stabilized, the usual interval between visits and lithium serum measurements in young adults goes from 8 to 12 weeks, but elderly patients are monitored more frequently. Routine visits for lithium patients include: (a) medical evaluation; (b) psychiatric evaluation; (c) completion of a checklist of side effects and concurrent medications; (d) measurement of serum concentrations of lithium 12 ± 1 h after the last dose. On admission and on a yearly basis thereafter, we require routine laboratory tests, thyroid function tests, electrocardiogram, and a visit by a cardiologist. Our therapeutic range for lithium maintenance in elderly patients is widened to 0.40–0.80 mmol/l compared to the 0.60–0.80 mmol/l range indicated for younger adults.

For the purpose of the present study, we extracted the following variables from patients’ charts: demographic characteristics, psychiatric diagnosis, history of maintenance treatments prior to lithium, current lithium dose, concurrent medications, and data on renal and thyroid functions.

Patients were followed up for 6 years (last evaluation, August 31, 2016), with particular emphasis on status of lithium treatment, concurrent medications, and thyroid and renal functions.

### Renal function

Serum creatinine concentrations were taken from the panel of laboratory tests requested on an annual basis. The traditional standardization method for serum creatinine was used.

The estimated Glomerular Filtration Rate (eGFR) was calculated from serum creatinine values using the equation proposed by the Modification of Diet in Renal Disease (MDRD) Study Group (Levey et al. 1999; Earley et al. 2012), with the ‘186’ correction factor, which also takes into account age, gender and ethnicity. The following categories of eGFR were considered: higher than 90 ml/min/1.73 m^2^ (G1); 60–89 ml/min/1.73 m^2^ (G2); 45–59 ml/min/1.73 m^2^ (G3a); 30–44 ml/min/1.73 m^2^ (G3b); 15–29 ml/min/1.73 m^2^ (G4); lower than 15 ml/min/1.73 m^2^ (G5). The abbreviations and ranges recall those used by KDIGO (2013) 2012 Clinical Practice Guidelines for the Evaluation and Management of Chronic Kidney Disease (CKD) (2012), but it must be noted that KDIGO CKD stages are also based on albuminuria categories, which were not included in the present study.

### Statistical analysis

We used the following statistical tests: (a) unpaired or paired Student’s *t* test to compare means; (b) nonparametric Mann–Whitney test to compare medians; (c) Fisher’s exact test to analyze contingency tables: (d) multiple regression analysis to study the effects on renal function of age, gender and years of lithium treatment at inception and during follow-up, using eGFR or eGFR decline as the dependent variable; (e) a generalized linear model for repeated measures to analyze the influence of age and gender on eGFR variation during the 6-year follow-up; (f) Cox regression analysis was performed to analyze time between the start of the study and achievement of eGFR <45 ml/min/1.73 m^2^, as a function of age, gender and duration of prior lithium exposure.

## Results

### Cross-sectional data at start

At the start of the study, median serum concentration of lithium in the 110 patients was 0.55 mmol/l. Patients were taking regularly the following medications: *N* = 80 (73%) benzodiazepines, *N* = 57 (52%) ACE inhibitors, sartans, and/or thiazides, *N* = 21 (19%) oral hypoglycemic agents or insulin, and *N* = 34 (31%) hypolipidemic agents.

Mean daily doses of lithium carbonate were significantly lower in patients taking ACE inhibitors, sartans, and/or thiazides (384 ± 187 versus 464 ± 196 mg; *P* = 0.03).

Regarding thyroid function, *N* = 33 (30%) were taking levothyroxine, and *N* = 2 (2%) were taking methimazole. With regard to renal function, 43 (39%) had an eGFR lower than 60 ml/min/1.73 m^2^.

Demographic and clinical characteristics of patients according to the age of their first lithium prescription are shown in Table 1.

**Table 1:** Demographic and clinical characteristics at baseline by age at first lithium prescription

	Patients who started lithium at age <65 (*n* = 79)	Patients who started lithium at age ≥65 (*n* = 31)	*P*
Female/male (% females)	59/20 (75%)	21/10 (68%)	0.48
Age, median (min–max)	70 (65–84)	77 (65–83)	0.00006
Years on lithium, median (min–max)	18 (0–34)	7 (0–16)	2 × 10^−15^
Referred by neurologist	4 (5%)	14 (41%)	0.0001
Lithium carbonate daily dose, median mg (min–max)	450 (150–1050)	450 (150–750)	0.48
DSM-5 diagnosis			
Bipolar I disorder	36 (46%)	7 (23%)	0.03
Bipolar II disorder	22 (28%)	12 (39%)	0.36
Major depressive disorder, recurrent	18 (23%)	2 (6%)	0.06
Bipolar disorder NED (not elsewhere defined)	3 (4%)	10 (32%)	0.0001
Current psychotropic medication			
Anticonvulsants	15 (19%)	1 (3%)	0.04
Antipsychotics	33 (42%)	6 (19%)	0.03
Antidepressants	18 (23%)	4 (13%)	0.30
Benzodiazepines	55 (70%)	25 (81%)	0.34
Prior long-term antidepressants	1 (1%)	8 (26%)	0.0001
Availability of brain imaging (CAT or NMR scans)	12 (15%)	10 (32%)	0.06
Antiplatelet drugs or oral anticoagulants	14 (18%)	13 (42%)	0.01
ACE inhibitors, sartans, and/or thiazides	40 (51%)	17 (55%)	0.83

After correcting for the effects of age and gender, duration of lithium treatment was associated with lower eGFR values (Table 2).

**Table 2:** Multiple regression analysis of eGFR at baseline

Variable	Beta	*P* value	95% Confidence interval	
Gender (male versus female)	3.86	0.34	−4.05	11.77
Current age	−0.10	0.74	−0.69	0.49
Years of lithium treatment	−0.85	1.82 × 10^−6^	−1.19	−0.52

Compared to the remaining patients, the subgroup (*N* = 31) starting lithium for the first time during old age (≥65 years) included greater proportions of patients with the following characteristics: *N* = 14 (41%) had been referred to the lithium clinic by a neurologist; *N* = 10 (32%) had a diagnosis of bipolar disorder not elsewhere defined; *N* = 8 (26%) had previously been exposed to chronic antidepressants; *N* = 10 (32%) had undergone neuroimaging (CAT or NMR); and *N* = 13 (42%) were taking antiplatelets or oral anticoagulants.

### Follow-up

**Fig. 1:**
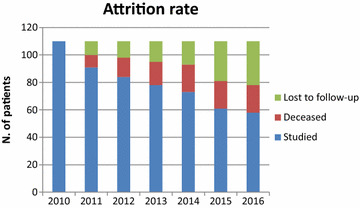
Attrition rate of patients during 6-year follow-up. Number of patients studied, deceased, or otherwise lost to follow-up over the 6 years of the study

At the end of the 6-year follow-up, 58 (53%) patients were still on lithium (Fig. 1), including 7 patients who had continued lithium but were not able to come into the clinic because of physical disability. Of the remaining patients, 32 (29%) were lost to follow-up, while 20 (18%) had died due to intervening diseases (Fig. 1).

Death occurred at a median age of 76 years for the following causes: 6 malignancies, 2 respiratory diseases, 6 cardiovascular diseases (including 5 cerebrovascular), 2 renal diseases, 3 neurologic diseases, and 1 complications of diabetes.

Two patients were prescribed levothyroxine and two methimazole for the first time during follow-up.

In the 48 patients (9 men and 39 women) who had serum creatinine measured at the end of follow-up, eGFR had declined from a mean of 66.4 to a mean of 52.6 ml/min/1.73 m^2^ (Student’s *t* test for paired samples; *P* = 7 × 10^−9^), corresponding to an annual decline of 2.3 ml/min/1.73 m^2^. The generalized linear model for repeated measures confirmed that eGFR decreased from 66.4 ml/min/1.73 m^2^ (95% CI 61.0–71.8) to 52.6 ml/min/1.73 m^2^ (95% CI 47.5–57.9) during the 6-year follow-up, corresponding to a mean variation of 13.8 ml/min/1.73 m^2^ (95% CI 9.8–17.7). The model did not detect effects of age and gender, but the small sample size is to be taken into account. In a Cox regression analysis, incidence rate of eGFR <45 increased by 0.61% (95% CI 0.17–1.06%) for each year of prior lithium exposure, with no influence of age and gender.

## Discussion

This observational study provides a snapshot of current prescription attitudes regarding lithium treatment in the elderly at our center.

### Dosing and maintenance of serum concentrations

Maintenance doses in this elderly cohort (median 450 mg of lithium carbonate daily) were much lower compared to the usual dosage of 900–1500 mg of lithium carbonate per day used in younger adults (Baldessarini and Tarazi 2006). Such doses are consistent with the results from the McGill Geriatric Lithium-Induced Diabetes Insipidus Clinical Study (Rej et al. 2014b) reporting that the lithium dose required to achieve a given serum concentration decreases threefold from middle to old age. With regard to the therapeutic range, serum concentrations are not well established in the elderly. In our lithium clinic, the lower end of the range for elderly patients is reduced to 0.40 mmol/l compared to the 0.60 mmol/l generally indicated for younger adults (Bauer and Gitlin 2016), principally to prevent episodes of lithium toxicity associated with reduced renal function, drug interactions, and risk of dehydration, all of which are typical of old age.

Moreover, there is evidence that the ratio between serum and brain concentration may decrease with age (Moore et al. 2002).

### Comedication

The high proportion of patients from this cohort taking regularly psychiatric and non-psychiatric medications warrants some comments. For example, it is perhaps surprising that 3/4 of our patients were on long-term benzodiazepines. It is possible that the specialists prioritized the need to control anxiety and/or sleep disorders over the widely known detrimental side effects of benzodiazepines on cognition (Larson et al. 1987). Another point of note is that medications that are theoretically contraindicated in lithium patients (ACE inhibitors, sartans, and thiazides) were taken regularly by more than half of the patients in this cohort. The likely explanation here is that hypertension must be treated even in lithium patients, provided that the doses are reduced adequately. This notion is corroborated by the fact that patients in this cohort treated with ACE inhibitors or sartans were taking significantly lower lithium doses. Moreover, with regard to cases with reduced eGFR, the decision to treat patients with ACE inhibitors or sartans was often taken by the nephrologist, based on the evidence that these drugs may slow the progression of CKD (Ruggenenti et al. 2012). We have not included data on other nephrotoxic drugs, namely NSAIDs, because they were not taken regularly but only when needed.

### Diagnoses

A subgroup of patients in this cohort who began taking lithium when elderly recalls the case series of 10 patients described by Hagop Akiskal’s group of “elderly patients with late-onset mood and related behavioral symptomatology and cognitive decline without past history of clear-cut bipolar disorder”. The authors reported that “symptoms were often refractory to or aggravated by antidepressants…, whereas mood stabilizers and/or atypical antipsychotics were beneficial, promoting behavioral improvement in all treated patients and marked cognitive recovery in five”. Only one case had been treated with lithium (Ng et al. 2008). We did not measure cognition in the present study, but the above-mentioned subgroup had a history of behavioral dysregulation, mood lability, and cognitive symptoms that improved after withdrawing long-term antidepressant treatment and starting lithium. According to referring specialists, their choice of prescribing lithium was also based on its neuroprotective properties (Bauer et al. 2003). Interestingly, a longer duration of lithium treatment has been reported to be related to higher white matter integrity among elderly patients with bipolar disorder (Gildengers et al. 2015). In another study using voxel-based morphometry, Zung et al. (2016) found an increased left hippocampal volume in the lithium-treated group compared with the nonlithium-treated group, and decreased left hippocampal volume in the nonlithium group relative to controls.

### Thyroid function

One-third of patients from this cohort were taking thyroid hormone replacement therapy, including two patients who were prescribed levothyroxine for the first time during follow-up. It has long been known that lithium is associated with hypothyroidism, and that middle-aged women are at higher risk (Johnston and Eagles 1999). In the aforementioned cross-sectional study of thyroid function by Kraszewska et al. (2015), of 45 women receiving long-term lithium (mean age, 63 years), seven (16%) were receiving levothyroxine replacement therapy. In three patients, this drug had been introduced within the 1st year of lithium therapy, and in the remaining four patients after 8, 11, 12, and 33 years.

Hyperthyroidism requiring antithyroid medication was less frequent in patients from this cohort (4%), and manifested for the first time during follow-up in two cases.

Therefore, we conclude that thyroid function continues to require close monitoring throughout lithium treatment.

### Renal function

The problem of lithium-associated renal dysfunction has recently been addressed by several research studies and reviews (Rej et al. 2012, 2015). With regard to old patients, a series of studies from the University of Toronto have provided several pertinent results. In a 4-year retrospective cohort study of 42 patients, lithium levels did not correlate with change in eGFR, suggesting that levels up to 0.8 mmol/l are safe in geriatric patients without pre-existing chronic renal failure (Rej et al. 2013a). In a 5-year retrospective cohort study of 27 geriatric patients with eGFR lower than 60 ml/min/1.73 m^2^, changes in eGFR in patients who continued lithium for at least 2 years and those who discontinued lithium did not differ significantly. However, clinically important decreases in eGFR occurred in the majority of continuers but in none of the discontinuers (Rej et al. 2013b). In a 4-year retrospective cohort study of 82 patients, geriatric psychiatry patients were found at higher risk for clinically important decreases in eGFR than 200 psychotropic-naïve similarly aged controls. Multivariate analyses of potential risk factors for renal dysfunction (including age, hypertension, diabetes, diuretics, and duration of lithium treatment), suggested that lithium is an important factor when eGFR is lower than 60 ml/min/1.73 m^2^ (Rej et al. 2014c). In a population-based cross-sectional study of 2480 lithium users aged ≥70 years, lithium use for >2 years was one of the factors independently associated with CKD, together with hypertension, diabetes mellitus, ischemic heart disease, nephrogenic diabetes insipidus, acute kidney injury, and use of loop diuretics, hydrochlorothiazide, or atypical antipsychotics (Rej et al. 2014b).

We have previously reported that duration of lithium treatment is to be added to advancing age as a risk factor for reduced glomerular filtration rate (Bocchetta et al. 2013, 2015). In a retrospective regression analysis of the last available eGFR regarding 953 (596 women, 357 men) patients of any age, eGFR was found lower in women (by 3.47 ml/min/1.73 m^2^), in older patients (0.73 ml/min/1.73 m^2^ per year of age), and in patients with longer lithium treatment (0.73 ml/min/1.73 m^2^ for each year) (Bocchetta et al. 2015). In the present study, the effects of sex and age were not significant because of the small sample size and the limited age range.

The decline attributable to lithium was 0.85 ml/min/1.73 m^2^ per year of prior exposure. During follow-up, the decline was faster, corresponding to an annual decline of 2.3 ml/min/1.73 m^2^. It must be noted that 39% of patients had already an eGFR lower than 60 ml/min/1.73 m^2^ at baseline, and the initial mean eGFR in patients followed up for 6 years was 66.4 ml/min/1.73 m^2^. There are data suggesting that, once CKD is established, eGFR further declines irrespective of lithium withdrawal (Bendz et al. 2010; Bocchetta et al. 2015). A similar pattern of decline is also observed in the general population: for example, a longitudinal analysis performed on 4074 subjects from the Sardinia study cohort (Pani et al. 2014) revealed that eGFR declined by 1.87 ml/min/1.73 m^2^ per year in individuals with a baseline eGFR of <60 ml/min/1.73 m^2^ compared to a decline of 0.80 ml/min/1.73 m^2^ per year in individuals with a baseline eGFR of ≥60 ml/min/1.73 m^2^.

In a 2-year randomized, placebo-controlled trial followed by single-blind extension, lithium treatment was not associated with renal dysfunction (Aprahamian et al. 2014). The trial was carried out in the context of a study of long-term lithium treatment for amnestic mild cognitive impairment in the elderly (Forlenza et al. 2011). It must, however, be noted that the target of lithium concentrations was low (0.25–0.50 mmol/l), and subjects with CKD might have been excluded from the trial, because enrollment was reliant on the approval of the general practitioner.

A recent population-based study concluded that, after adjustment for several confounders (including comorbidities, co-prescriptions, and episodes of lithium toxicity), the decline attributable to lithium was not significant (Clos et al. 2015). However, this study did not include patients older than 64 years, and patients with an eGFR <60 ml/min/1.73 m^2^ represented only 1% of their cohort.

### Limitations of the study

The noninterventional nature of this study implies several limitations. Apart from ordinary clinical procedures, no specific instruments were used to evaluate response to treatment or side effects. Cognition was not measured, and neuroimaging was not performed systematically. With regard to the role of lithium exposure in the decline of renal function, the absence of a control group has to be taken into account. Moreover, several potential risk factors for CKD (such as hypertension, diabetes, co-medications) that can be detected in population-based samples were not adjusted for because of the small size of this clinical sample.

## Conclusions

This observational study provides some clues regarding lithium treatment in the elderly. As comorbidity and polypharmacy may increase the risk of adverse events and drug interactions, median lithium serum concentration in this cohort was lower than the therapeutic range indicated for younger adults. Decline in glomerular filtration rate may be accelerated by long-term lithium use. Thyroid and renal functions continue to require close monitoring throughout lithium treatment.

## Abbreviations

eGFR: estimated glomerular filtration rate
CKD: chronic kidney disease
